# SEE-TREND: SEcurE Traffic-Related EveNt Detection in Smart Communities

**DOI:** 10.3390/s21227652

**Published:** 2021-11-18

**Authors:** Stephan Olariu, Dimitrie C. Popescu

**Affiliations:** 1 Department of Computer Science, Old Dominion University, 3300 Engineering & Computational Sciences Bldg., Norfolk, VA 23529, USA; olariu@cs.odu.edu; 2 Department of Electrical and Computer Engineering, Old Dominion University, 231 Kaufman Hall, Norfolk, VA 23529, USA

**Keywords:** smart cities, smart communities, smart mobility, congestion support, cyber–physical systems

## Abstract

It has been widely recognized that one of the critical services provided by Smart Cities and Smart Communities is Smart Mobility. This paper lays the theoretical foundations of SEE-TREND, a system for Secure Early Traffic-Related EveNt Detection in Smart Cities and Smart Communities. SEE-TREND promotes Smart Mobility by implementing an anonymous, probabilistic collection of traffic-related data from passing vehicles. The collected data are then aggregated and used by its inference engine to build beliefs about the state of the traffic, to detect traffic trends, and to disseminate relevant traffic-related information along the roadway to help the driving public make informed decisions about their travel plans, thereby preventing congestion altogether or mitigating its nefarious effects.

## 1. Smart Cities and Smart Communities

This introductory section is intended to offer a succinct review of Smart Cities and Smart Communities. Specifically, Section 1.1 reviews key concepts related to Smart Cities; Section 1.2 reviews a few attributes of Smart Communities; finally, Section 1.3 takes a look at smart mobility, one of the fundamental services provided by Smart Cities and Smart Communities.

As a help to the reader, Table 1 provides the definitions of all acronyms used in the paper.

### 1.1. Smart Cities

Visionaries have invented the Smart City metaphor almost 30 years ago to give expression to the idea that urban communities were benefiting from the most recent advances in technology, innovation, and globalization [1]. Recently, the National Academies of Sciences, Engineering, and Medicine [2] defined Smart Cities as “urban centers that use intelligent, connected devices and automated systems that maximize the allocation of resources and the efficiency of services”.

Inspired by recent urbanization reports, the United Nations predicted that, if the current trends continue, by 2050, well over 68% of the world population will live in metropolitan areas [3]. In the light of this, and of the above-mentioned urbanization reports, the relevance of Smart Cities will increase tremendously.

The visionaries have defined Smart Cities in myriad different ways [4,5,6,7,8], As it turns out, however, these definitions have three characteristics in common:

- First, the Smart Cities will adopt an anticipatory governance style aligned with the real needs of the citizens;
- Second, they provide their citizens with timely access to high-quality information and services;
- Third, they rely on a broad-based participation of the citizens in the co-creation, in partnership with the local government, of services needed by the community.

These two fundamental characteristics of Smart Cities can be summarized by saying the Smart Cities will “put their citizens first” or, equivalently, that they will be citizen-centric communities [9]. The citizen-centric characteristic of Smart Cities is aligned with the open e-government services proposed in the past two decades by the European Union [10] and permeates the work of [11,12]. Importantly, providing their citizens with unrestricted access to high-quality information and services will naturally lead to the co-creation of new services aligned with the needs and wants of the citizenry [9,13].

The transition from the urban communities of today to the Smart Cities of the near future, predicted by the visionaries, will be enabled by the incredible technological advances that we are witnessing every day. One of the challenges involved in this transition is to understand how ICT can be used to empower the people, protect the environment, take action is response to climate change, attend to the needs of all segments of the population, support and promote economic vitality, etc. [13,14,15]. By leveraging ICT, the Smart City will deliver a large array of “Smart Services” including, *Smart Government* [10,11,12], *Smart Healthcare* [16,17], *Smart Homes* [18,19], *Smart Mobility* [20], *Smart Parking* [21], *Smart Transportation* [22,23], and *Smart Workplaces* [24], among many similar ones.

Recently, the authors of [9] have drawn attention to the fact that, in a very strong sense, Smart Cities are Cyber–Physical Systems wherein the Physical component is represented by the deployed infrastructure represents the Physical, while the Cyber component involves the people, their administration, and the apps they use. The Cyber and the Physical components of the CPS are locked in a dialectic relationship where they feed, condition, and learn from each other. An interesting corollary of this is that the Cyber and Physical components thrive or fail together. Consequently, the Smart Cities of the near future will continually evolve and enhance the services they offer to the citizens. This will lead to the evolution of diverse Smart Cities based on their own characteristics and the needs of their citizens. Furthermore, of course, this is confirmed by the challenge of running Smart Cities. Should the Cyber and Physical components of a Smart City run away from each other, the entire entity is bound to suffer. In recent years, we have witnessed a large number of urban communities call themselves Smart Cities. Many of these communities were Smart Cities in name only because their two components did not work together well.

It is reasonable to expect the Smart Cities of the near future to provide the socio-economic foundation for sustainable material well-being and prosperity based on an advanced service platform. This will be provided through various forms of cooperation and co-work ranging from human-to-human, to human-to-machine, and eventually to machine-to-machine [25,26]. Most of these forms of cooperation are novel, especially those arising between humans and machines or between autonomous machine systems, have yet to be adequately formalized and understood [14,27,28,29].

### 1.2. Smart Communities

In a recent solicitation [30], the U.S. National Science Foundation described Smart Communities as “*communities that synergistically integrate intelligent technologies with the natural and built environments, including infrastructure, to improve the social, economic, and environmental well-being of those who live, work, or travel within it*”. The NSF definition of a Smart Community is consistent with the vision and stated goals of Society 5.0 recently publicized by the Japanese Government [13]. Aligned with this definition, Eltoweissy et al. [14] argued that a Smart Community should be motivated by the goal of satisfying most of the reasonable needs of their citizens through the co-creation and provisioning of high-quality services. Eltoweissy et al. [14] pointed out that in a Smart Community the resources and services will be valuated through a *Marketplace of Goods and Services* that will act as an impartial arbiter between producers and consumers of services. Unlike a Smart City that assumes a geographical co-location, a Smart Community is only logically co-located and not geographically. The vision expressed by [14] was to take the idea of utility computing to the next level of generality. Specifically, ref. [14] envisioned future Smart Communities as offering a large variety of evolving services packaged and sold as utilities and managed by a marketplace.

### 1.3. Smart Mobility—A Key Service in Smart Cities and Smart Communities

We anticipate that the transition for present-day communities, whether urban or otherwise, to the Smart Cities and Smart Communities of the near future, will offer challenging opportunities for the creative and efficient use of all the available or contemplated resources and services. Among the services offered by Smart Cities and Smart Communities, *Smart mobility* is, and in all likelihood will remain, one of the most challenging to provide in a coherent and sustainable fashion. Smart mobility involves the creative use of the latest advances in ITS in order to make traffic more fluid, better adapted to the needs of the driving public while, at the same time, minimizing its societal and environmental footprint. For example, Smart mobility should be designed in an inclusive rather than exclusive way. Smart mobility should elevate the pedestrian and cyclist experience, making them first-class citizens [22,31,32].

Smart Transportation has two key components, namely *Smart Mobility* [6] and *Smart Parking* [33,34]. In a very recent paper, Aljohani et al. [33] devoted their attention to Smart Parking solutions in Smart Cities. Their goal was to identify, among the many published papers dealing with parking, innovative solutions for parking-related problems that can be employed in the Smart Cities of the near future. In this paper, we are devoting our attention exclusively to Smart Mobility solutions that make traffic more fluid and more efficient, elevating the experience of the driving public. Specifically, we show how SEE-TREND can provide innovative solutions to Smart Mobility.

## 2. SEE-TREND—Motivation

Statistical data for 2019 (the last year for which data are currently available) originating with the US-DOT and NHTSA show convincingly that, contrary to popular belief, 45% of all traffic fatalities were attributable to highway traffic-related incidents rather than to rush-hour traffic in big cities [35,36]. In addition to the loss of life and property caused by traffic accidents, the US-DOT has shown that traffic-related congestion results in 2.9 billion gallons of wasted fuel, 5.5 billions hours of wasted productivity, and is also responsible for the release into the atmosphere of more than 56 billion pounds of CO${}_{2}$ [37]. Not surprisingly, reducing the number of traffic incidents and making our roadways safer and more environmentally friendly is recognized as one of the key challenges of Smart Cities and Smart Communities [21,38].

Since on most American roadways traffic volumes are close to capacity, congestion is a common occurrence triggered by random fluctuations in traffic flow [37]. Traffic engineers have argued that given sufficient advance notification of traffic flow *trends*, drivers could make educated decisions about taking alternate routes, thus reducing congestion, saving time and drastically curtailing CO${}_{2}$ emissions [39,40,41,42]. Unfortunately, no mechanism for detecting traffic trends well in advance is available at the moment [43,44].

In order to promote Smart Mobility in the Smart Communities of the near future, there is a critical need to *detect* trends in traffic flow, including premonitory signs of imminent slowdown and congestion. Such a system would allow drivers to make more informed decisions about their travel, therefore preventing congestion altogether or mitigating its nefarious effects.

To address this critical need, the main *objective* of this paper is to lay the theoretical foundations of SEE-TREND: a system for Secure Early Traffic-Related EveNt Detection in Smart Cities and Smart Communities. SEE-TREND implements an anonymous, probabilistic collection of traffic-related data from passing vehicles. The collected data are then aggregated and used by its inference engine to build beliefs about the state of the traffic, to detect traffic trends, and to disseminate relevant traffic-related information along the roadway.

SEE-TREND was designed to investigate issues related to the integration of wireless networking with lightweight roadside infrastructure into a CPS that enables efficient, secure, and privacy-preserving detection of traffic-related events and the timely dissemination to the driving public of relevant information about them. The key novelties of SEE-TREND are that, in sharp departure from existing approaches, our system:

1. Detects existing traffic conditions and discovers discernible trends in the traffic flow; this allows SEE-TREND to predict imminent traffic events and to alert interested parties to their likely occurrence;
2. Intelligently disseminates relevant traffic-related information using a recently-developed information decay model. This model developed by [45], captures the rate at which the value of traffic-related information decays as a function of time and distance;
3. Is secure, privacy-aware and non-intrusive. Being non-intrusive, the deployment of SEE-TREND will not adversely affect the durability and structural integrity of our roadways, as ILDs do.

### Our Contributions

SEE-TREND is a transformative, next-generation distributed system that will help promote sustainable Smart Mobility is the context of the Smart Cities of the near future. SEE-TREND will contribute to aggregating traffic-data collected by the huge fleet of vehicles on our roadways into a comprehensive, near real-time synopsis of traffic conditions nationwide.

Our contributions can be summarized as follows:

- We have developed techniques for setting up and managing secure data collection. SEE-TREND collects traffic-related data from vehicles participating in the traffic flow. Since, in general, these data are highly correlated, there is no need to collect data from *all* passing cars, especially in dense traffic. In support of privacy and security of data collection, every message will be associated *anonymously* with a physical vehicle, eliminating the need for IDs or pseudonyms;
- SEE-TRENDS detects trends in the traffic flow by aggregating the collected data: SEE-TREND aggregates traffic flow data collected from participating vehicles and uses the resulting information to *anticipate* trends in the traffic flow and to infer the likely occurrence of traffic events including imminent slowdowns and congestion;
- SEE-TRENDS enables efficient traffic-related information dissemination. SEE-TREND provides two integrated modalities for information dissemination. For non time-critical information dissemination, SEE-TREND relies on vehicles in the oncoming traffic; however, time-critical messages are disseminated directly by the SEE-TREND roadside infrastructure. One of our key objectives is to further extend the model of information decay both in time and distance [45]. Such a model can be used to optimize the dissemination of relevant traffic-related information thus reducing network traffic;
- SEE-TRENDS supports information dissemination in planned evacuations and emergencies. SEE-TREND allows emergency management personnel to disseminate important information to drivers. This feature of the system is vital during large-scale evacuations, allowing emergency managers to alert drivers to estimated travel times, to the availability of resources, and to contraflow policies.

This paper is organized as follows: in Section 1 we begin by offering a short introduction to Smart Cities, Smart Communities, and Smart Mobility. Next, Section 2 motivated SEE-TREND as a key catalyst for enabling Smart Mobility. Section 3 offers a review of technical issues that will facility the presentation. Specifically, in Section 3.1 we present our vehicular model; in Section 3.2 we introduce vehicular networks and the wireless communication model assumed throughout the paper; in Section 3.3 we discuss briefly security issues in vehicular networks; in Section 3.4 we introduce Intelligent Transportation Systems; in Section 3.5 we review smart-phone based systems for detecting traffic-related events. Next, in Section 4 we outline the architecture of SEE-TREND. Specifically, in Section 4.1 we introduce the TMU, the work-horse of SEE-TREND; Section 4.4 discusses the coverage area of TMUs. Section 4.2 discusses secure TMU to TMU communications. This is followed by Section 4.3 that discusses secure vehicle to TMU communications and by Section 4.5 discussing role-based vehicle-to-TMU Communications. Next, Section 4.6 discusses a probabilistic data collection strategy. Following this, Section 4.7 and Section 4.8 discuss, respectively, how to make SEE-TREND secure and fault-tolerant. Section 5 discusses various strategies that SEE-TREND employs to disseminate traffic-related information to the driving public. Specifically, Section 5.1 presents the dissemination of non-time critical information. This is followed, in Section 5.2 by strategies if time-critical information dissemination; further, in Section 5.3 we discuss role-based information dissemination, while in Section 5.4 strategies for information dissemination in planned evacuations is discussed. Section 6 presents a case study of how SEE-TREND can be used for the early detection of potholes on highways. Finally, Section 7 offers concluding remarks and identifies challenges ahead.

## 3. State of the Art

SEE-TREND straddles a number of research areas ranging from exploiting vehicular on-board resources, to vehicular networks, to ITS, and to security and privacy, to name just a few. The main goal of this section is to offer a succinct synopsis of relevant recent developments in these areas, focusing particularly on previous work on which we build.

### 3.1. The Vehicle Model

We assume that vehicles have the following on-board capabilities:

- GPS receiver;
- A short-range, DSRC-compliant, wireless transceiver;
- A tamper-resistant Event Data Recorder (EDR). The EDR is responsible for recording mobility attributes including acceleration, deceleration, lane changes, etc. [46]. Each such transaction is associated with an instantaneous GPS reading (in its 2006 ruling [47], the NHTSA has mandated that starting September 2010 an EDR will have to be installed in vehicles with an unloaded weight of less than 5000 lbs). All of the vehicle’s sub-assemblies feed their readings into the EDR [48,49].

With millions of new cars sold in the US each year [35], we will soon have millions of these advanced vehicles on our roadways and city streets.

### 3.2. Vehicular Networks

The original impetus behind VANET, a variant of MANET, was to provide the driving public with real-time notification of traffic and road conditions. The promise of VANET has intrigued the wireless networking research community over the past three decades. Over the years, a number of applications were developed, various standards were written, and many useful protocols were proposed and implemented.

In the past three decades a number of VANET prototypes have been deployed in Europe and Japan by consortia involving car manufacturers [50,51,52], government agencies and various standardization bodies [53,54]. In the US, the PATH initiative has investigated fundamental issues ranging from platooning, to various forms of automated driving, and to collision warning systems [39,41]. Recently, the US-DOT inspired by the reality of present-day vehicles endowed with a vast panoply of on-board sensors and powerful radios, has launched the Connected Vehicles Initiative [55,56] whose stated goal is to investigate the integration of ITS with vehicular communications. The idea behind the Connected Vehicles Initiative is that connected vehicles, using V2V and/or V2I communications, could have a tremendous potential on traffic monitoring and incident detection [57,58,59].

### 3.3. Security and Privacy Issues in VANET

Privacy and security issues are of fundamental importance in VANET. The bulk of security solutions proposed for VANET use standard cryptographic techniques that rely on encryption to promote trust between communicating vehicles or between vehicles and the roadside infrastructure with which they are communicating. Recently, a number of protocols have been developed for key agreement supporting confidentiality and authentication services in VANET [41,60,61]. There seems to be a consensus in the Cyber-security community that while symmetric-key-based protocols tend to be simpler, they are not sufficiently flexible for use in dynamically reconfigurable networks. Public key-based protocols offer more flexibility and scalability but tend to be slow and to consume a great deal of computational resources. In order to avoid vehicle identification, several approaches advocate the use of time-varying pseudonyms. To implement this solution, vehicles need to contact periodically either roadside infrastructure or a certificate authority to obtain new pseudonyms, or preload many pseudonyms into a tamper-proof on-board device [62,63]. However, even with the use of pseudonyms, tracing communication to the owner of the vehicle is often difficult to prevent [64,65,66,67].

### 3.4. Intelligent Transportation Systems

One of the main goals of ITS is to offer the driving public a host of traffic state monitoring and data collection facilities. This is achieved by using a combination of legacy technologies and more recent initiatives leveraging V2V and/or V2I communications and sensor technologies [68,69,70,71,72]. It is fair to say that the past decade has witnessed a convergence of vehicular networking technology with ITS, promising to revolutionize the way we drive by creating a safe, secure, and robust ubiquitous and pervasive computing environment that will eventually encompass our highways and city streets [37,73].

Well known traffic monitoring and incident detection techniques proposed in the past forty years, many of which are still in use today, employ ILDs, video detection systems, acoustic tracking systems and microwave radar sensors [74,75,76]. Of these, the most prevalent are the ILDs used to measure traffic flow by registering a signal each time a vehicle passes over them. Each ILD, including hardware and controllers, costs around USD 8200; in addition, adjacent ILDs are connected by optical fiber that costs USD 300,000 per mile [77]. Unfortunately, official statistics show that, at any moment in time, over 50% of the installed ILD base and 30% of the video detection systems are defective or otherwise unavailable [78,79]. Moreover, by the extensive use of wireless communications have raised security and privacy concerns, very similar to those encountered in VANET. Not surprisingly, transportation departments worldwide are looking for more reliable and more cost-effective solutions for traffic monitoring and incident detection [37].

### 3.5. Smartphone-Based Systems

The widespread adoption of smartphones has suggested enlisting these and other similar portable devices such as iPads, FitBits, Smart Watches, Smart Glasses and the like to supplement legacy traffic monitoring by allowing the driving public to provide input in the form of ad hoc incident reports. An early implementation of this idea has resulted in *511 Traffic* intended to offer an at-a-glance view of road conditions in a given geographic area [80]. However, one of the key limitations of 511 Traffic is that it is a *centralized* system that accumulates and aggregates traffic-related feeds at TMCs. Due to inherent delays, 511 Traffic often displays out-of-date traffic information [81].

The recently-completed Mobile Millennium project at the University of California Berkeley harvested and exploited traffic-related information collected by probe vehicles to infer information about the state of the traffic [82]. A lesson learned from the Mobile Millennium project is that relying solely on traffic data collected by probe vehicles seems to work best in urban environments that experience a high concentration of vehicles and less well on highways where the number of probe vehicles is smaller [83].

Finally, Google maps is a GPS-based geographical navigation application program for smartphones with GPS support and display screens which provides traffic status information from driver-submitted location-dependent traffic status information along with travel times and route details. Waze is a centralized system where the entire communication takes place over the mobile telephone network.

All smartphone-based systems known to us rely on the existence of a predeployed cellular phone systems. As a result, these systems may fail to work when they are most needed, that is, in the case of emergencies, whether human-made or natural, where some of the infrastructure may no longer operational. Such is the case, for example, in evacuation scenarios in the wake of a hurricane, a terrorist attack, an earthquake and the like [28,84]. In comparison, SEE-TREND is energetically self-sufficient and does not rely on predeployed infrastructure support.

## 4. The Details of SEE-TRENDS

This section presents the details of SEE-TREND and discusses the integration of its various components.

### 4.1. The TMU—The Workhorse of SEE-TRENDS

**Figure 1:**
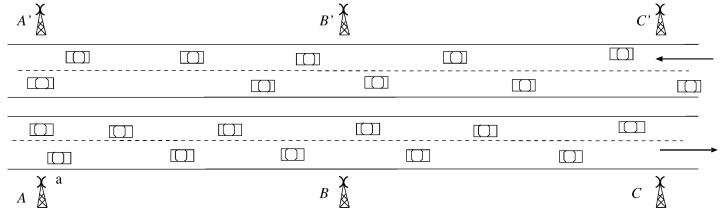
Illustrating a highway segment with several TMUs [85] ©IEEE.

The workhorse of SEE-TREND are lightweight roadside TMUs that are positioned along the roadway at uniform distances (e.g., every kilometer or so) and are designed to be independent of the power grid, with minimal maintenance and service requirements. Furthermore, adjacent TMUs along the roadway are not connected with each other, which will result in significant cost savings when compared to the existing ILD technology that requires expensive optical fiber for interconnecting the ILDs. Figure 1 presents for illustration a roadway segment with three TMUs, *A*, *B* and *C* in one direction paired with $A^{\prime }$, $B^{\prime }$, $C^{\prime }$ in the opposite direction.

Each TMU contains an embedded low-power computing device with a GPS and a radio transceiver, and is powered by an energy harvesting system consisting of solar panels and a battery pack that ensures the TMU energy self-sufficiency [86]. Assuming that the TMU is powered by a 12 V/200 Ah battery that holds a total of 2400 Wh of energy, and that it consumes about 15–20 W, the energy required for daily operation does not exceed 480 Wh of energy in a 24 h period. This leads to a daily battery discharge of about 20%, and the energy required to fully recharge the battery can be obtained from a 0.9 m${}^{2}$ 100 W solar panel (or two 50 W panels) with as little as 4 h of sunlight per day [87,88]. Moreover, with current rechargeable batteries supporting approximately 2000 charging cycles over their lifetime, the 20% per day discharge rate of the battery translates into a 6+ years of battery life, which represents a conservative estimate based on existing battery and solar panel technologies [89,90]. We note that, for practical implementations, more accurate calculations are necessary, which should consider additional details that are beyond the scope of the paper, such as the reliability of the solar energy for example, which is dependent on the geographic location of the system, weather patterns, as well as other factors.

As the central components of SEE-TREND the TMUs collect and aggregate traffic-related data from passing vehicles, as well as by exchanging information, intermittently, with adjacent TMUs. When they become aware of confirmed traffic *trends*, the TMUs disseminate this information to the vehicles that travel on the roadway. When no vehicles are present and no traffic information needs processing, the TMUs enter in a low-power vigilant mode where they only transmit periodic identification information to alert approaching vehicles, which will result in additional power savings extending the lifetime of the TMU battery.

### 4.2. Secure Data Exchange between Adjacent TMUs

For normal operation, adjacent TMUs are not expected to exchange information directly, and they rely instead on passing vehicles to carry information from one TMU to the other when the traffic conditions are normal. However, whenever time-critical information needs to be exchanged between adjacent TMUs, they have the ability to communicate directly for short periods of time using a DSRC-like radio interface that covers distances up to 1 km [91].

To secure the communication link between them, adjacent TMUs, say *A* and *B*, along the roadway (see Figure 1) share a *time-varying* symmetric key μ(A,B,t) that is used to encrypt, at time *t*, the data exchanged between them, and using GPS synchronization, the TMUs switch from one key to the next in a pre-established order based on their local time. Thus, SEE-TREND implements a robust scheme that will allow adjacent TMUs to communicate securely.

To illustrate the data exchange between TMUs *A* and *B* shown in Figure 1, let us consider that a non time-critical message *m* must be transmitted from *A* to TMU *B*. The process will start with the encryption of message *m* by TMU *A* with μ(A,B,t) followed by upload of encrypted message *m* onto passing vehicle *a*. When *a* arrives at TMU *B*, it will drop off message *m* for decoding by *B*. If TMU *B* has to send a message to TMU *C*, it would apply a similar process using a symmetric key μ(B,C,t), known only to *B* and *C*. We note that if the key chain contains a sufficiently large set of keys, the encryption keys used for TMU to TMU communication will appear random to an external observer.

**Figure 2:**
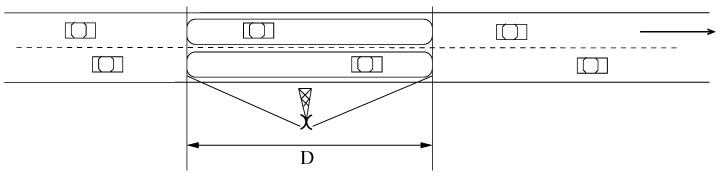
Illustrating the coverage area of a TMU [85] ©IEEE.

The TMU radios will use multiple access technology to communicate with vehicles traveling in different lanes that are within the coverage area of a TMU at the same time, as shown in Figure 2. In this instance, distinct lanes in the TMU coverage area will be assigned non-interfering radio channels to allow vehicles in adjacent lanes to communicate with the TMU in parallel. The use of multiple access schemes also enables the TMU to communicate with several vehicles that are in the same lane within the TMU coverage area. We note that the length *D* of the TMU coverage area depends on various factors that are discussed in Section 4.3, and indicate that for vehicles traveling speeds of 70 mph a value of *D* = 45 m should be sufficient and may be covered by available short-range wireless technologies such as ZigBee or UWB [92,93,94,95,96].

### 4.3. Secure Vehicle to TMU Communication

A vehicle approaching a TMU is either allowed to drop off its collected EDR data with the TMU or else is considered to be a “new” vehicle and is not authorized to do so. To be more precise, those vehicles that have correctly handshaken with the previous TMU have received a one-time session key α that allows them to drop off their EDR data, upon correctly handshaking with the next TMU. On the other hand, those vehicles that just entered the highway or have failed to handshake with the previous TMU are considered “new” vehicles and are not allowed to drop off their EDR information with the TMU. Since, by assumption, the TMUs are synchronized, a TMU can easily check for validity an alleged session-key α. It is easy to see that using one-time session keys issued by the previous TMU, precludes malicious vehicles (including rogue vehicles stationed by the roadside) from mounting a credible Sybil attack on the TMU. Furthermore, it is important to note that the session key is independent of the identity of the vehicle (and driver). In effect, this allows for *privacy-aware* communications between passing vehicles and TMUs.

**Figure 3:**
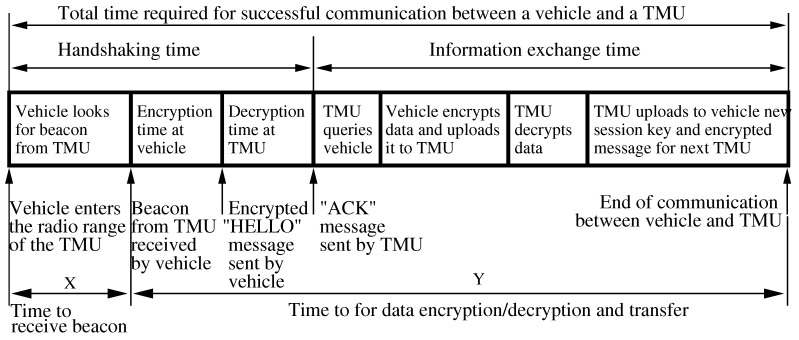
Illustrating the details of vehicle to TMU communication [85] ©IEEE.

As illustrated in Figure 3, a connection between a vehicle and a TMU is established when the vehicle enters the TMU’s coverage area and receives, on a control channel λ, a beacon containing handshaking information [85]. Having received the handshaking information, the vehicle answers with a “HELLO” message containing

- λ encrypted with the one-time key α, or else
- Only λ if the vehicle is “new” and does not have a session key α.

The TMU then replies with an “ACK” message either encrypted with α or else issues to the vehicle a one-time session key β representing the exact contact time encrypted with the symmetric key between the current and next TMU down the road.

Consider a vehicle that was issued a one-time key β. When this vehicle reaches the next TMU it will upload β to the TMU and, upon successful validation, will be allowed to drop off data collected by its EDR. As a precautionary measure, the TMU verifies that the time at which the vehicle passed the previous TMU as recorded by its EDR corresponds to the value β properly decrypted. If the two match, the credentials are accepted and the data exchange proceeds. Otherwise, the credentials are rejected and the vehicle is, again, considered “new” and is not allowed to drop off EDR data. We note that even “new” vehicles are entitled to download any notification and warning messages that the TMU has available. However, since they are not trusted entities, they are not allowed to drop off EDR data with the TMU.

Next, consider a vehicle whose one-time session key α is recognized as valid. The “ACK” message returned by the TMU contains a random frequency channel σ (encrypted with α) on which subsequent data exchange is to take place; in addition, a secure one-time key may be established for the purpose of the data exchange. The vehicle switches to channel σ and proceeds to the data exchange with the TMU by:

- First, dropping off its EDR data along with encrypted data from the previous TMU;
- Second, downloading any notification and warning messages that the TMU has available.

It is important to note that the data exchange between vehicle and TMU requires that a wireless radio link be established and that data be successfully transmitted. The former requirement depends on the TMU beacon spacing (that is how often the beacon signal is sent by the TMU) and on the time required to encryption/decryption the “HELLO” message sent by the vehicle. The latter requirement depends on the encryption/decryption times and the data rate of the wireless link.

### 4.4. Reasoning about the TMU Coverage Area

It should be clear that successful information exchange between vehicles and TMUs depends on the amount of time the vehicles spends within the coverage area of the TMUs. For simplicity, we assume that vehicles travel at a constant speed *s* and that each TMU has a coverage area of size *D* meters. Using the notation in Figure 3 let the random variable *X*, with distribution function *F*, keep track of the time between the moment a vehicle enters the TMU coverage area and the time it receives the first beacon. We assume that *X* is uniformly distributed in $\left[0,\frac{D}{s}\right]$. Similarly, let the random variable *Y*, with *general* distribution function *G*, keep track of the time it takes to perform the information exchange between the vehicle and TMU. We further assume that *X* and *Y* are independent.

Now, the probability of the event, $P_{success}$, that the information exchange is successful is described by the conditional distribution of X+Y given that the event $\left\{X\leq \frac{D}{s}\right\}$ has occurred. By the assumed independence of *X* and *Y*, we have that

(1) $$\begin{array}{ccc} P_{success} & = & \mathrm{Pr}\left[X+Y\leq \frac{D}{s}\;|\;X\leq \frac{D}{s}\right]\\ & = & \int_{0}^{\frac{D}{s}}P\left[X+Y\leq \frac{D}{s}\;|\;X=u\right]\;dF\left(u\right)\\ & = & \frac{s}{D}\int_{0}^{\frac{D}{s}}\mathrm{Pr}\left[Y\leq \frac{D}{s}-u\right]\;du. \end{array}$$

Assuming that *X* has a uniform distribution in the interval $\left[0,\frac{D}{s}\right]$ and that *Y* has a general distribution G(·) expression (1) can be written as

(2) $$\begin{array}{ccc} P_{success} & = & \frac{s}{D}\int_{0}^{\frac{D}{s}}G\left(\frac{D}{s}-u\right)\;du=\frac{s}{D}\int_{0}^{\frac{D}{s}}G\left(x\right)\;dx.\; \end{array}$$

**Figure 4:**
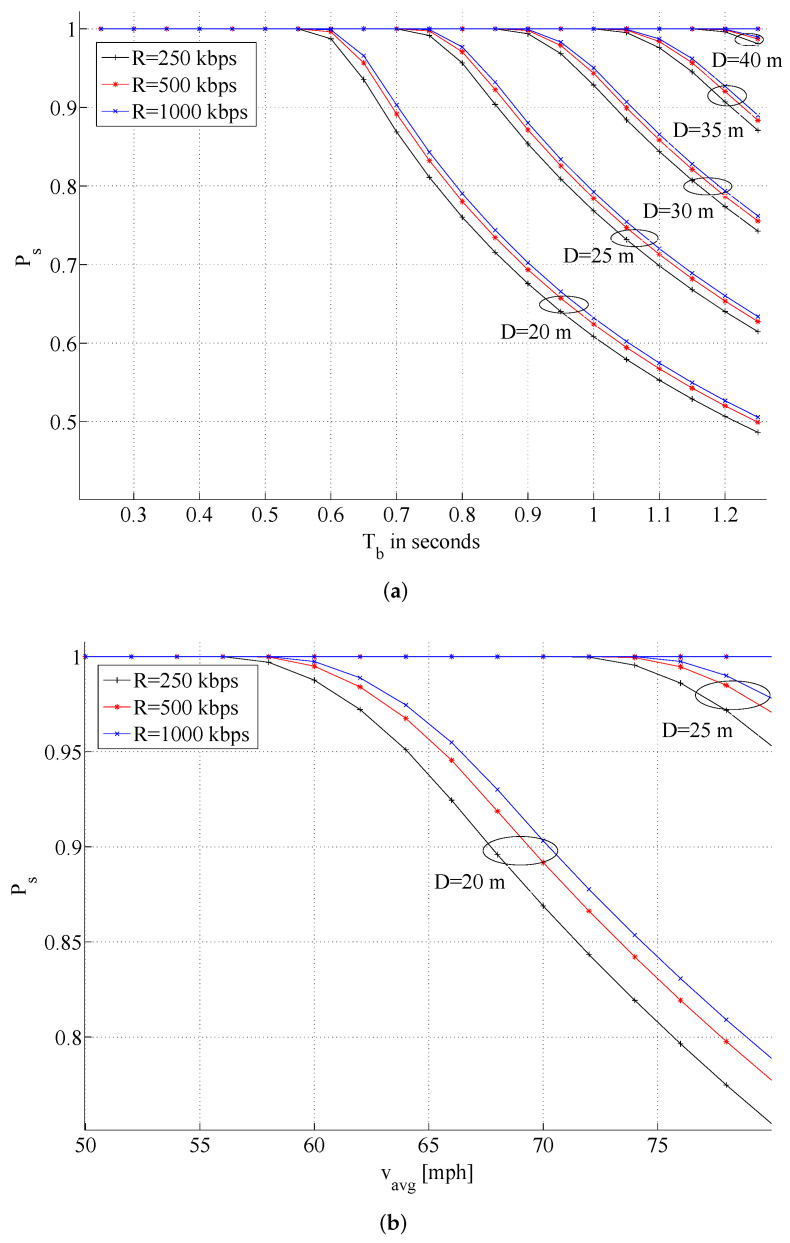
Illustrating $P_{success}$ for various data rates *R* and coverage distances *D*. (**a**) Varying beacon interval with $v_{avg}=70$ mph, (**b**) Varying average vehicle speed with $T_{b}=0.7$ s.

To get a handle on $P_{success}$, we have considered the worst-case scenario where the maximum amount of information $I_{\max}$ is always exchanged between a vehicle and the TMU and have experimented with the following estimates (see Figure 3): 8 kb maximum data transferred between vehicle and TMU at data rates, *R*, of 250, 500, and 1000 kbps; $Y\left(y\right)=I_{\max}/R$ for all *y*; beacon intervals $T_{b}$ starting from 100 ms (typical for 802.11-based wireless systems) to around 1.1 s; 50 ms for encrypting and decrypting a 1 kbyte “HELLO” message. Using these values we calculate $P_{success}$ given in Equation (2) and plot its values in Figure 4, from which we note that a value of D≃45 m ensures $P_{success}\approx 1$ for data rates achievable with various short-range wireless technologies. The TMU coverage distance can be reduced if the requirement that the TMU successfully exchange information with all vehicles is relaxed. For example, when $P_{success}=0.8$ (i.e., the TMU exchanges information with 80% of the passing vehicles) the TMU coverage distance *D* can be reduced to about 30 m or less.

### 4.5. Role-Based Vehicle to TMU Communications

In special situations, communications between SEE-TREND and a number of authorized vehicles such as police cruisers, ambulances, fire-fighter trucks and the like is necessary. In these cases, the communication between TMUs and passing vehicles needs to be augmented to allow authorized vehicles to interact with SEE-TREND in a predetermined, *role-based*, fashion. As mentioned, this is essential to the interaction with first responders including ambulances, fire fighters, and local police. To accommodate these special needs, authorized vehicles using a special encryption key will be allowed to upload essential information to individual TMUs. For example, police cruisers may upload information related to various types of alerts, planned lane closures, contraflow, suggested detour routes, as well as the availability of essential resources at various interchanges [97].

### 4.6. Enabling Probabilistic Data Collection

Recall that SEE-TREND becomes aware of traffic-related conditions by collecting EDR data from passing vehicles. Not surprisingly, these data are highly correlated simply because the vehilces in a local cohort see roughly the same traffic conditions. Thus, there is no need to collect data from *all* passing vehicles, especially in dense traffic, and one of the important issues we will investigate is *probabilistic data collection*.

Assume that, instead of collecting data from every passing vehicle, the TMU tosses a biased coin that turns up heads with probability *p* and collects data from a given passing vehicle with probability *p*. Assume, further, that the data collected from *k* vehicles leads to the correct detection of a raffic-relayed event with probability $1-e^{-\theta k}$, for some application-dependent parameter θ>0 [98,99]. Let $A_{n}$ be the event that, under the assumptions above, *n* passing vehicles suffice for the purpose of detecting correctly a traffic event.

Let *H* be the random variable that keeps track of the number of vehicles that have contributed traffic-data among the *n* passing vehicles. By conditioning on the event *H* we have

(3) Pr[An]=∑k=0nPr[An|H=k]Pr[H=k]=∑k=0nnkpk(1−p)n−k1−e−αk=∑k=0nnkpk(1−p)n−k−∑k=0nnkpe−αk(1−p)n−k=1−1−p1−e−αn.

Of course, the value of *n* depends on the vehicle arrival rate which, in turn, depends on the traffic flow. If a particular TMU is aware of the intensity of the traffic flow, it can determine the number *n* of arrivals per unit time. In turn, this will allow the computation of *p*. In fact, the TMU can deliberately forgo data collection from a number of vehicles provided the traffic flow intensity permits. This will reduce TMU power consumption without impacting the time to detect a traffic-related condition.

### 4.7. Implementing Security Solutions in SEE-TREND

By design, SEE-TREND allows anonymous data collection while preventing impersonation attacks. We now describe possible approaches to providing security against confidentiality and DoS attacks.

SEE-TREND provides built-in security against confidentiality attacks. This is because, as discussed in Section 4.3, the handshaking protocol between a TMU and a passing vehicle involves encrypted data and is not intelligible to the adversary, even positioned by the roadside. An intrinsic part of the handshake is the establishment of a frequency on which subsequent communication between the TMU and the passing vehicle will be conducted. This, ensures that the subsequent data exchange is not understood by the adversary.

Even with these security safeguards in place, a number of problems remain open and will be investigated in this proposal. DoS attacks can take several forms and mitigating their effects is one of the key challenges of SEE-TREND. One form of DoS attack occurs when two adversaries, *X* and *Y*, working together, each positioned by the roadside close to adjacent TMUs *A* and *B* attempt the following scheme: *X* will pick up the all the keys β that TMU *A* provides to “new” vehicle and transmits them to *Y*. *Y* then will transmit to *B* pretending to be a valid vehicle. TMU *B* will detect that the use of β is fraudulent (since the time stamp does not match); when the vehicle that was issued key β attempts to contact TMU B the attempt will be rejected since the one-time key has already been used by the adversary. If the number of “new” vehicles is large, this form of DoS attack may result in artificially thinning out the traffic seen by TMU *B* and, as a result, delaying the time it takes SEE-TREND to detect an event.

### 4.8. Making SEE-TREND Fault-Tolerant

**Figure 5:**
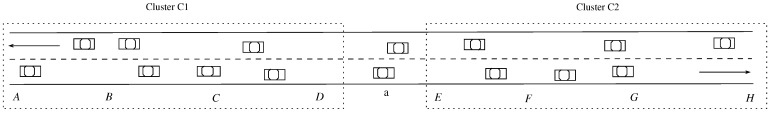
Illustrating clustering and fault tolerance in SEE-TREND.

Another important issue in SEE-TREND is fault tolerance. The basic questin here is this: how can the functionality of SEE-TREND be preserved when a TMU is disabled as a result of a malfunction or power failure. Consider the two-lane highway segment shown in Figure 5 and assume a vehicle *a* traveling between TMUs *D* and *E* which share the time-varying symmetric key μ(D,E,t) used to encrypt, at time *t*, all the messages carried between the two. If *E* is disabled, the messages uploaded by *D* are lost, since they cannot be decoded by the next active TMU, *F*. This, in effect, renders vehicle *a* “new”, which may adversely impact the timeliness of data collection in SEE-TREND.

We now discuss A possible approach to providing fault tolerance in SEE-TREND. The idea is to induce a simple cluster structure on the set of TMUs by grouping k,(k≥2), adjacent TMUs into a cluster, as shown in Figure 5. We shall refer to *k* as the *clustering factor*. In this clustering scheme, every TMU belongs to exactly one cluster and, therefore, the resulting clusters are disjoint. The idea is that as long as each cluster contains an active TMU, messages can be correctly propagated end-to-end in accord with the SEE-TREND semantics. The clustering of the TMUs can be static or dynamic. In either case, each TMU is informed about the identity of the cluster to which it belongs. Figure 5 illustrates two adjacent clusters, with clustering factor k=4 where TMUs A,B,C,D belong to cluster C1 while TMUs E,F,G,H belong to C2.

In the clustered version of SEE-TREND, all TMUs in cluster $C_{i}$ know two keys, $\mu (C_{i-1},C_{i},t)$ and $\mu (C_{i},C_{i+1},t)$. Symmetric key $\mu (C_{i-1},C_{i},t)$ is used to encrypt, at time *t*, messages originating in cluster $C_{i-1}$ and destined for some TMU in clusters $C_{i-1}$ or $C_{i}$. Symmetric key $\mu (C_{i},C_{i+1},t)$, used to encrypt, at time *t*, messages originating in cluster $C_{i}$ and destined for some TMU in clusters $C_{i}$ or $C_{i+1}$.

Referring again to Figure 5, vehicle *a* carries a message encrypted by TMU *D* with key $\mu (C_{1},C_{2},t)$. If TMU *E* is active, then upon reaching this TMU, the messages is properly encrypted and a new (or the same) message encrypted with the key $\mu (C_{2},C_{3},t)$ is uploaded onto *a*. However, if *E* is disabled, then *a* fails to establish communication with *E* and carries the message to the next TMU, *F*. Assuming that *F* is active, it correctly decrypts the message and uploads to *a* a new message encrypted with key $\mu (C_{2},C_{3},t)$ informing subsequent TMUs in the same cluster that *E* is disabled. Observe that key $\mu (C_{2},C_{3},t)$ must be used since *F* does not know the number of remaining active TMUs in its own cluster.

To determine the amount of fault tolerance afforded by our clustering scheme let us consider a SEE-TREND consisting of *n* consecutive TMUs with a clustering factor of k,(1≤k<n). A good measure of fault tolerance is the probability of the event *X* that SEE-TREND will fail to propagate messages end-to-end. To evaluate this probability, we assume that TMUs fail independently of each other with some technology-dependent probability *p* and look at the sequence of *n* TMUs as a string σ of *n* symbols *A* and *D*, with *A* denoting an active TMU and *D* representing a disabled one. Assuming all nk possible arrangements of σ equiprobable (i.e., adopting the Fermi–Dirac statistics [100] (p. 54)), the probability of a given *k*-TMU cluster to be disabled is nk−1. Thus, P[X]=⌊nk⌋nk−1 and the probability that an entire cluster is down is $p^{k}$, which implies that the probability that there is no end-to-end connection is given by

(4) ∑i=1nknkipki(1−pk)nk−i=1−1−pknk≈1−e−npkk.

For example, for n=30, k=4 and p=0.1, this probability evaluates to 0.0749%.

Having presented the components of SEE-TREND, in the next section we describe how SEE-TRENDS realizes its stated objectives.

## 5. Enabling Efficient Traffic-Related Information Dissemination in SEE-TREND

SEE-TREND provides two integrated modalities for information dissemination that depend on the nature of information to be disseminated, which can be non time-critical or time-critical. The latter refers to information that needs to be disseminated urgently to allow the driving public to make timely decisions, as is the case with information about a crash, for example, which, when suitably disseminated, allows drivers to take alternative exits sparing them the trouble of being stuck in traffic waiting for the crash to clear. We note that, for non time-critical information dissemination, SEE-TREND relies on vehicles in oncoming traffic, while time-critical messages are disseminated directly by TMU to TMU communication. We begin by discussing the dissemination of non time-critical information such as travel time estimates, traffic flow statistics, average speed, road work information as well as minor congestion.

### 5.1. Non Time-Critical Information Dissemination

**Figure 6:**
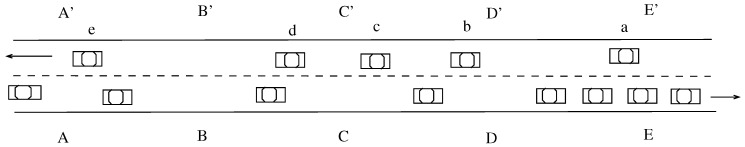
Illustrating non time-critical information dissemination on a two-lane roadway.

For the dissemination of non time-critical traffic information SEE-TREND uses vehicles as data mules to relay encrypted traffic information between consecutive TMUs. Once they receive the relevant information, the TMUs will share it with the passing vehicles, as discussed in Section 4.3. We use Figure 6 to illustrate the backwards information propagation of non time-critical information and note that, by aggregating data from passing vehicles, TMU *E* becomes aware of a minor slowdown and disseminates this information to TMUs, A,B,C,D which, in turn, inform the passing vehicles. Referring to Figure 6, TMU *E* begins by passing the information to TMU $E^{\prime }$. Next, TMU $E^{\prime }$ uploads onto vehicle *a* slowdown information destined for TMU $D^{\prime }$. Once TMU $D^{\prime }$ learns about the slowdown it will inform *D* and will also upload the information onto vehicle *b*. The same process is then continued until TMU *A* receives the information. Finally, TMUs A,B,C,D inform passing vehicles about the slowdown so that they can take appropriate action.

### 5.2. Time-Critical Information Dissemination

**Figure 7:**
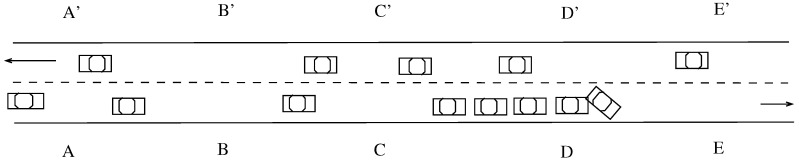
Illustrating time-critical information dissemination on a two-lane roadway.

In order to be effective at reducing incident-related congestion, SEE-TREND must be able to quickly disseminate information to the vehicles that have not yet reached the incident area. Referring to Figure 7 imagine that TMU *E* sees a sudden significant drop in traffic volume. At the same time, TMU *D* sees stopped or crawling traffic. The other TMUs still see free flowing traffic. It is essential to determine that an accident has occurred between TMUs *D* and *E*. One approach for accomplishing this is the following. Upon seeing a sudden drop in traffic, TMU *E* sends a query to TMU *D* on a control channel. TMU *D* sees a significant increase in traffic volume and a significant reduction in speed and is also tuning in to the control channel. It is important to note that the other TMUs are not yet aware of the accident and they do not tune in to the control channel. Now it is a simple manner for TMUs *D* and *E* to establish that an accident must have occurred between them. This information is time-critical and will be disseminated to the other TMUs.

### 5.3. Enabling Role-Based Information Dissemination

At various times, emergency management personnel and police officers need to provide drivers with important information. Since SEE-TREND is, at its most basic level, an information dissemination tool, it seems natural to allow these personnel to insert information into the system. As mentioned earlier, authorized vehicles will be given special encryption keys, used to facilitate communication with the TMUs. One of the most frequent uses of the role-based mode is when police have verified a traffic accident and are driving to the scene. As they pass TMUs, the police can input additional information about the accident that will be provided to drivers approaching the accident, validating and corroborate the findings in Section 6.

### 5.4. Supporting Information Dissemination in Planned Evacuations and Emergencies

As already mentioned, SEE-TREND is engineered to allow first responders and other traffic management personnel to disseminate important information to drivers. This feature is vital during large-scale evacuations, allowing emergency managers to alert drivers to estimated travel times, to the availability of resources, and to the implementation of various contraflow policies [97].

In order to minimize impact of human lives and property, massive evacuations are often necessary in the face of predictable natural disasters such as hurricanes and tsunamis. However, it is well known that there are several issues involved in a large-scale evacuation. For example, once an evacuation is underway, finding resources, such as gasoline, drinking water and shelter, quickly becomes an issue [101,102]. In two reports to Congress on hurricane evacuations [103,104] the US-DOT found that emergency evacuation plans often do not consider availability of such resources. The US-DOT also determined that emergency managers need a method for communicating with evacuees during the evacuation in order to provide updated information. The reports cited above suggested that traffic monitoring equipment should be deployed to provide real-time traffic information along evacuation routes. Aligned with this need, SEE-TREND can provide travel time estimates, notification of available resources, such as gasoline, food, and shelter, and notification of contraflow.

To facilitate this type of monitoring and information dissemination using SEE-TREND, emergency officials can place temporary hardware in the form of support infrastructure, sturdy tamper-proof devices, or mini-towers in the median, about every 10 miles in advance of an evacuation. It is important to note that these devices are temporary and would only be deployed in an emergency. The temporary devices would plug into the TMU system and connect SEE-TREND to the emergency management center overseeing the evacuation. This would provide the TMUs with important information that cannot determined solely by SEE-TREND. In addition, these devices can use powerful radios send backward messages in the case of contraflow traffic, where all lanes travel in the same direction, away from the evacuation zone.

Using the temporary hardware, SEE-TREND can query vehicles about travel times and upload that information to the emergency management center. This vital travel time information could then be released to the public via the news media (TV and radio) and the Internet, which would allow potential evacuees to make informed decisions about if and when to leave the area. In addition, this aggregated information would be fed back into the TMU system to give drivers the same information as they pass over the TMUs.

To determine open gas stations, the EDR monitors the gas tank and determines when gas was added. In addition, the vehicle knows how far it has traveled since the last fill-up. The TMU closest to each highway on-ramp can query traffic to see when and where they last added gasoline. SEE-TREND could then use this information to inform travelers of nearby available gas stations. To facilitate the dissemination of additional information, state emergency agencies could require that gas station operators, hotel operators, and restaurants who remain open during the evacuation provide accurate information to the central server, which would then provide this information to drivers via SEE-TREND. In this same way, emergency managers can upload information about open shelters to the central server. It is important to note that this system would be used to facilitate an evacuation before a disaster strikes, so we assume that electricity and network connections are available.

In addition to having state and county authorities send information to the TMUs about evacuations or contraflow policies, using role-based communication as described earlier, the TMUs themselves could determine the direction and speed at which traffic is flowing. Drivers entering on-ramps onto contraflow roadways (these ramps would likely have been used as exit ramps previously) could be alerted to the direction the traffic is moving. TMUs on one roadway could also alert drivers to upcoming on-ramps that were previously used as exit ramps during non-contraflow travel. As an additional feature, since the TMU system can monitor traffic flow, SEE-TREND could offer recommendations for which roadways are being heavily traveled in only one direction. These roadways are likely good candidates for contraflow.

## 6. A Case Study: Pothole Detection

Current traffic monitoring techniques perform some amount of Automated Incident Detection (AID) using established ITS algorithms [105,106,107,108,109]. Most of the algorithms, though, are threshold-based, meaning that traffic must be monitored for some amount of time to determine what “normal” is. After that, conditions that are different from “normal” are flagged as incidents. Unlike existing systems which use standard traffic models that describe normal traffic intensity variations to detect significant changes in traffic intensity, we plan to use the unique communications between TMUs to develop AID algorithms that are self-calibrating. In this direction, we note that SEE-TREND aggregates traffic-related data collected from passing vehicles and uses this data to detect traffic-related events and to anticipate trends including imminent slowdowns and congestion. For example, TMUs can monitor the speed of passing vehicles and share that information with neighboring TMUs. A TMU detecting a sudden drop in the average speed, but not in the volume of traffic, can infer that congestion is imminent. We propose to study the use of a Distributed Expectation Maximization (DEM) algorithm that performs local computations at each TMU and passes a small set of sufficient statistics to neighboring TMUs in the iteration process.

For a preliminary analysis let us consider *M* TMUs and the *m*-th TMU records $N_{m}$ speed measurements $\left(y_{m,1},\ldots ,y_{n,N_{m}}\right)$. Let N denote the Gaussian density function with mean μ and covariance σ. The measurements are assumed to follow a Gaussian mixture distribution of the form

(5) $$y_{m,i}=\sum_{j=1}^{J}\alpha_{m,j}N\left(\mu_{j},\sigma_{j}\right),\;i=1,\ldots ,N_{m}$$

where the mixing parameters $\left\{\alpha_{m,j}\right\}$ are potentially unique at each TMU, but the means $\left\{\mu_{j}\right\}$ and covariances $\left\{\sigma_{j}\right\}$ are common at all TMUs. The goal of the proposed DEM algorithm is to estimate these parameters from the data $y=\{y_{m,i}\}$.

For j=1,…,J, we define $\phi =\{\mu_{j},\sigma_{j}\}$ as the set of means and covariances, $\alpha_{m}=\left\{\alpha_{m,j}\right\}$ as the mixing probabilities for each TMU *m* and $\theta =\phi ⋃{\left\{\alpha_{m}\right\}}_{m=1}^{M}$. The DEM algorithm computes a Maximum-Likelihood (ML) estimate, i.e., θ maximizing the log-likelihood function

(6) $$L_{y}\left(\theta \right)=\sum_{m=1}^{M}\sum_{i=1}^{N_{m}}\log\left(\sum_{j=1}^{J}\alpha_{m,j}N\left(y_{m,i}|\mu_{j},\sigma_{j}\right)\right)$$

where N(y|μ,σ) denotes the evaluation of a Gaussian density with mean μ and covariance σ. We note that our measurements are highly correlated and hence the maximum-likelihood can be interpreted as a “pseudo-likelihood” whose estimates tend to the true ML estimates as the data tends to infinity [110].

The estimated mean, variance, and mixing parameters mirror the characteristics of the traffic intensity in a quantitative manner. In the case of congested roads (lower speed distributions), the mean quantifies the severity of congestion and for un-congested roads (higher speed distributions), the mean may be used to define the acceptable/normal traffic intensity. The measure of variance represents the consistency in traffic intensity while the mixing parameters represent the congestion index on a scale of 0 to 1.

We propose a probabilistic technique to detect both temporary incidents (e.g., accidents, disabled vehicles, etc.) and long-lived anomalies (e.g., potholes) using vehicle-to-TMU communications. The proposed technique, which is the first of its kind to detect such traffic-related events, starts with some *beliefs* about the road, such as assuming that the probability of having an incident has a very small value.

When SEE-TREND notices a number of lane changes due to temporary incidents and/or abnormal road conditions, the corresponding data collected will be correlated in both time and location, and will provide *evidences* for the system to update beliefs by computing the *posteriori* probability using Bayesian inference techniques with the arrival of each *evidence* as

(7) $$Bel\left(Incident\right)=\frac{P(incident)*P(Evidence|Incident)}{P(Evidence)}=\alpha *P\left(incident\right)*P\left(Evidence|Incident\right)$$

where P(incident) is the *a priori probability* of an incident at a given location, P(Evidence|Incident) is the likelihood, α can be computed as

(8) α=1.0/[P(Incident)∗P(Evidence|Incident)+P(NoIncident)∗P(Evidence|NoIncident)]

and, finally, Evidence represents any evidence such as changing lane or passing over a pothole.

Accumulated empirical evidence has confirmed that in order to avoid a lane occlusion, most vehicles change lanes at a moderate distance away from it and that, under most traffic conditions, the distance to the occlusion at which the lane change occurs is *normally distributed*. We formally define the conditional probability of a lane change at location *x* given that an occlusion exists at location *y* as

(9) $$P_{x,y}=\frac{1}{\sqrt{2\pi }}e^{\frac{-{\left(x-y\right)}^{2}}{2}}.$$

On the other hand, drivers may notice potholes at the last minute and hence we expect them to change lanes, if at all, very close to the pothole. Thus, we define the conditional probability of a lane change at location *x* given that a road condition exists at location *y* as

(10) $$P_{x,y}=\frac{I(A=true)}{\sqrt{2\pi }}e^{\frac{-{\left(x-y\right)}^{2}}{2}}$$

where I(A=true) is an indicator function returning 1 if the driver had the ability to change lanes and *x* was near *y*, and 0 otherwise. This reflects the fact that some drivers may pass over potholes and not change lanes.

Each TMU maintains a list ManMade storing information about human-made road “anomalies” including rail-road crossings, speed ramps and road work. In addition, each TMU maintains two tables TempProb[m][n] and PermProb[m][n] where *m* is the number of lanes and *n* is the number of *segments* between two consecutive TMUs. Here, a segment is an atomic unit of length, say, around 10 m. The purpose of these tables is to record the probabilities of a temporary (respectively permanent) incident at any given segment. For example, TempProb[i][j]=0.8 indicates that there is a probability of 0.8 of having some form of a temporary occlusion of lane *i* at segment *j* between two given TMUs, where all probabilities were initialized to some very small value representing the normal probability of having an incident on that road.

Define those segments that a vehicle has traversed as “passed over segments” and the others as “avoided segments”. When a TMU receives an EDR report from a passing vehicle, it applies Bayes’ theorem to update the posterior probabilities for different road segments, for both tables, based on the new EDR data as follows.

For each segment p=(i,j) that a vehicle *x* has passed over, the following steps are performed.

- If *x*’s EDR has a record for a suspected road section at location p,p∉ManMade, then we need to compute the a posteriori probability of having an actual road condition at this location, update our beliefs, as
  (11) PermProb[i][j]=α∗PermProb[i][j]∗P(Detection|Pothole)
  where α is computed as before. The term P(Detection|Pothole) is industry–dependent and reflects how the probability of successfully detecting a road condition given that one actually exists.
- If *x*’s EDR shows that *x* has significantly decelerated around location *p*, p∉ManMade, this means that there might be a pothole at location *p* that the driver wanted to avoid or reduce its effect on their vehicle.
  (12) PermProb[i][j]=α∗PermProb[i][j]∗P(Reduce|Pothole)
  where α is computed as described before. The term P(Reduce|Pothole) represents the probability that a driver slows down when he sees a pothole. This depends on many factors including driver response.
- If *x*’s EDR shows nothing about *p*, then either *p* is clear or *x*’s driver avoided the pothole.

In all cases above, since *x* has passed over location *p*, there is no temporary incident at *p* and hence the probability of such an incident at this location should be re-initialized, i.e., we set TempProb[i][j]=InitialProbability. Next, assume that vehicle *x* has changed lanes at location y<j avoiding p(i,j). Observe that the lane change was due to a pothole, a temporary accident or a slow-moving vehicle. Thus, we need to compute the a posteriori probabilities as follows:

- First, we recompute the a posteriori probability of having a temporary incident at *p* that forced *x* to change lanes as $tempprob\left[i\right]\left[j\right]=\alpha *tempprob\left[i\right]\left[j\right]*P_{y,j}\;\left(0<i<y\right)$, where $P_{y,j}$ is the probability of changing lanes at location *y* given an incident at location *j* and can be computed as above;
- Furthermore, *x* may have changed lanes at *p*; we compute the a posteriori probability of having a pothole based on this new evidence: $permprob\left[i\right]\left[j\right]=\alpha *permprob\left[i\right]\left[j\right]*P_{y,j}$, (0<i<y), where $P_{y,j}$ is the probability of changing lanes at location *y* given a pothole at location *j* and can be computed as above.

Observe that the proposed technique cannot be deceived by slow-moving vehicles or other false positives.

After the TMU has processes data from a number of vehicles, if for some p(i,j)

- PermProb[i][j]>T for some threshold *T*, infer the presence of a pothole at location (i,j). The larger the value of the threshold *T*, the more conservative the TMU is;
- PermProb[i][j]=InitialPotholeProb and TempProb[i][j]>T, then infer the presence of a temporary incident at location (i,j) where InitialPotholeProb is the initial belief of having a temporary incident.

## 7. Concluding Remarks and Challenges Ahead

In this paper, we have laid the theoretical foundations of SEE-TREND, an innovative, next-generation computing and communication paradigm. We are confident that many other applications of SEE-TREND will be discovered which could have a profound and lasting societal impact. What makes SEE-TRENDS stand out, is that it aims to accomplish the following novel goals:

- First, our goal is to build a distributed system that detects existing traffic conditions and anticipates discernible trends in highway traffic flow. This allows our system to predict traffic events and to alert interested parties to their likely occurrence;
- Second, we will intelligently disseminate relevant traffic-related information using a decay model of the value of information based on time and distance;
- Third, SEE-TREND is a secure, privacy-aware, and non-intrusive system.

There are many ways in which SEE-TREND can be extended and enhanced. The most intriguing challenges include:

- Emergency vehicle preemption is one of the well-known causes of congestion. It is important to extend role-based interaction between emergency vehicles and SEE-TREND, while limiting the impact on traffic flow;
- Yet another intriguing question is: What is the most efficient way to manage contraflow, while reserving one incoming lane for ambulances, fire trucks, and the like?
- What are the tradeoffs between TMU energy and the quality of collected data in a planned evacuation?
- Fault tolerance is a key issue in emergencies and planned evacuations. We will investigate ways in which fault tolerance will be increased in support of such events.
- Extending SEE-TREND to detect traffic trends in urban settings, which may use alternative hardware such as traffic light controllers for example, instead of TMUs, and may employ strategies based on Vehicular Clouds [15,111] or Vehicular Crowdsourcing [29].

Furthermore, it would be very important to put to work Big Data analytics using computing resources that are available on-board modern vehicles. Big Data analytics in conjunction with Vehicular Crowdsourcing [29], Vehicular Clouds [15,111], and Machine Learning [112] could enable groups of vehicles to process more traffic-related data faster and more accurately. One of the promising directions is the potential for designing and implementing efficient hardware algorithms for basic operations including prefix computation, fast sorting and searching, as well as vision algorithms using various parallel architectures such as, for example, reconfigurable architectures [113,114,115,116] or parallel architectures with various fast bus systems [84,117,118,119,120,121]. Using parallel architectures to harvest Big Data analytics promises to extend the capabilities of SEE-TREND. This is a worthwhile area for future work.

## Figures and Tables

**Table 1:** A guide to the acronyms used in the paper.

Acronym	Description
AID	Automated Incident Detection
CPS	Cyber Physical System
DEM	Distributed Expectation Maximization
DSRC	Dedicated Short-Range Communications
EDR	Event Data Recorder
GPS	Global Positioning System
ICT	Information and Communications Technology
ILD	Inductive Loop Detector
ITS	Intelligent Transportation Systems
MANET	Mobile Ad hoc Networks
NHTSA	National Highway Traffic Safety Administration
PATH	California Partners for Advanced Transit and Highways
TMC	Traffic Monitoring Centers
TMU	Traffic Monitoring Unit
US-DOT	United States Department of Transportation
VANET	Vehicular Ad hoc Networks
V2I	Vehicle-to-Infrastructure
V2V	Vehicle-to-Vehicle
